# The use of nasal packing post rhinoplasty: does it increase periorbital ecchymosis? A prospective study

**DOI:** 10.1186/s40463-015-0075-5

**Published:** 2015-06-16

**Authors:** Ahmed M. Al Arfaj

**Affiliations:** Otolaryngology and facial plastic surgery consultant, college of medicine, King Saud University, P.O. Box 58588, Riyadh, 11515 Saudi Arabia

**Keywords:** Rhinoplasty, Septorhinoplasty, Ecchymosis, Packing, Complications

## Abstract

**Background:**

Periorbital edema and ecchymosis following rhinoplasty is disturbing for both the patients and their surgeons. The study aim was to determine whether nasal packing after lateral osteotomies in rhinoplasty surgery increases the risk of periorbital ecchymosis post-operatively.

**Methods:**

This was a prospective self-controlled single-blinded study. Seventy four patients who underwent rhinoplasty with bilateral lateral osteotomies by a single surgeon were enrolled in the study. Nasal cavity packing for one side was done while the other side was left unpacked. Periorbital ecchymosis was evaluated by the operating surgeon and a separate surgeon who is unaware of the packing side separately on the first, fourth and seventh day post-operatively. A 4-grade scale was utilized to assess the ecchymosis with grade 4 being the most severe.

**Results:**

Nasal packing was found to significantly increase the severity and duration of periorbital ecchymosis post rhinoplasty. While no difference was observed between the packed and unpacked sides on the first post-operative day, significant difference was noted on the 4th day (mean score 2.36 and 1.15 for the packed and unpacked sides, respectively) and on the 7th day after surgery in favor of the unpacked side (score 1.24 and 0.61 for the packed and unpacked sides, respectively).

**Conclusion:**

We advise against the routine use of nasal packing in rhinoplasty unless necessary as it contributes to worsen the periorbital ecchymosis from lateral osteotomies and thereby increases the patients’ “down time” after surgery.

## Background

In recent years, cosmetic surgery has been gaining more popularity in our modern world. This may be attributed to the increased safety profile of anesthesia techniques as well as improved outcome of cosmetic surgeries. Rhinoplasty in particular, is one of the most commonly performed cosmetic surgeries [1, 2] and as any surgical procedure, it has some well-documented side effects and complications [3]. Patient dissatisfaction rates range between 10 and 25 % and is highest in the early post-op period [4–6]. This can be partially attributed to nasal obstruction, facial edema, periorbital swelling and ecchymosis. Multiple studies have attempted to address periorbital ecchymosis by modifying surgical techniques, using anti-inflammatory agents such as steroids, using cold compresses, etc. [7–11].

The author of this article believes that following lateral osteotomies in rhinoplasty, the unnecessary use of nasal packing significantly contributes to increasing postoperative ecchymosis. This study aims to explore whether nasal packing has an effect on periorbital ecchymosis following osteotomies.

## Materials and methods

This was a prospective study conducted in King Abdul Aziz University Hospital, King Saud University, Riyadh, Saudi Arabia in the period between March 2014 and December 2014. Approval was obtained from the Institutional Review Board of King Saud University, College of Medicine. All patients who underwent rhinoplasty by a single surgeon were enrolled in the study. Only adult patients whose surgery required bilateral lateral osteotomies were included. In all cases, bilateral single low-to-low lateral osteotomies were performed endonasally using a sharp 4 mm guarded micro osteotome (Karl-Storz) 5 min after infiltrating the entry site with 0.5 ml of lidocaine 2 % with adrenaline 1:100,000. No periosteal elevation was performed prior to the osteotomies. Internal nasal splinting using polymeric silicone sheets (Silastic; Dow Corning) was performed in all cases, followed by nasal taping and dorsal splint application. A nasal pack was applied into one side of the nose, which was chosen randomly, while the other side was kept unpacked. The nasal pack used was a size 8 regular Merocele™ sponge (Medtronic: Metronic Xomed Inc FL, USA), which was removed 24 h post operatively. All patients were evaluated for periorbital ecchymosis by two surgeons separately on post-op day 1, 4, and 7. Patients who underwent other concomitant facial surgeries, needed multiple lateral osteotomies, underwent any turbinate surgery apart from simple outfracturing/radio frequency ablation, were known to have bleeding/coagulation disorders, using anticoagulants/antiplatelets (e.g. aspirin) or herbal supplements that may increase the risk of bleeding (e.g. garlic, vitamin E, gingko, etc.) along with those who had significant epistaxis intra or post-operatively that necessitated the use of nasal packing, were excluded from the study. None of the patients included in the study had medial, intermediate, or root osteotomies performed. Informed consent was taken from all patients as we do not practice nasal packing after septo/rhinoplasty unless significant bleeding was encountered. All surgeries were performed under general anesthesia which involved the use of fentanyl 2 mcg/kg, propofol 2 mg/kg IV and cisatracurium 0.15 mg/kg during induction. Anesthesia was maintained with a mixture of 40 % oxygen in air and sevoflurane gas. Patients received intermittent doses of cisatracurium (0.03 mg/kg) every 20–40 min as needed to maintain muscle relaxation. During the procedure, patients were placed supine, with head elevation to 30°. Normothermia was maintained throughout the procedure by using a warming blanket, intravenous fluid warmer, and heat and moisture exchanger. Mean arterial pressure (MAP) was maintained between 60 and 70 mmHg by varying the inspired sevoflurane concentration, additional doses of fentanyl (0.5 μg/kg) boluses and occasional labetalol 5 mg IV boluses to achieve the targeted MAP if needed. MAP drop < 60 mmHg was treated initially with reducing the end-tidal sevoflurane concentration, ephedrine 5 mg IV and intravenous fluids if needed. Dexamethasone, 8 mg IV was administered in all patients immediately after the induction of anesthesia. No further steroids were given during or after the procedure. Post-operatively all patients had their heads elevated to 30° for most of the first post-operative day. Ice packs were applied over the eyes intermittently for first 6 h following the procedure.

**Fig. 1:**
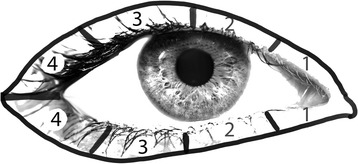
Grades of periorbital ecchymosis

On the 1st post-operative day, a surgical team member removed the nasal pack and 1 h later the operating surgeon and a different physician who is unaware of which nasal side was packed assessed and graded the periorbital ecchymosis separately using a 4-grade scale developed by Gürlek et al. [8] (Fig. 1).

Data was entered and analyzed using a computer based statistical package program (SPSS) V.22 (IBM, Armonk, NY). Chai-square was used to assess if there was a difference in ecchymosis severity within the same group, while Mann–Whitney *U* test was used to compare the severity of ecchymosis between the packed and unpacked side. For all statistical purposes, a *p* value of less than 0.05 was considered significant.

## Results

Seventy-four patients were included in the study, 54.1 % (*n* = 40) were males and 45.1 % (*n* = 34) were females. The participants’ average age was 25 ± 5 years with a range between 18 and 44 years. Forty-one patients (55.4 %) had external approach rhinoplasty, while 43 patients (44.6 %) underwent endonasal approach. All patients had one side of their noses packed intraoperatively. The nasal pack was placed on the right side in 37 patients (50 %) and on the left side in 37 patients (50 %). The grades of ecchymosis reported on 24 h, 4th and 7th day post-operatively are shown in Table 1. No major complications were reported in any of the patients. The reported postoperative complications consisted mainly of pain, nasal obstruction, peri-orbital swelling and ecchymosis (worse on the packed side). None of the cases has developed septal perforation, toxic shock syndrome or persistent septal deviation as a complication of nasal packing.

**Table 1:** A comparison of different grades of peri-orbital ecchymosis seen at day 1, day 4 and day 7 post rhinoplasty

Time post-op	Side	Grade 0	Grade 1	Grade 2	Grade 3	Grade 4	Mean Score	Standard deviation
Day 1	Packed	10 (13.5 %)	17 (23.0 %)	35 (47.3 %)	8 (10.8 %)	4 (5.4 %)	1.72	1.014
	Not packed	13 (17.6 %)	8 (10.8 %)	49 (66.2 %)	3 (4.1 %)	1 (1.4 %)	1.61	0.873
Day 4	Packed	4 (5.4 %)	6 (8.1 %)	32 (43.2 %)	23 (31.1 %)	9 (12.2 %)	2.36	0.987
	Not packed	24 (32.4 %)	20 (27.0 %)	26 (35.1 %)	3 (4.1 %)	1 (1.4 %)	1.15	0.975
Day 7	Packed	20 (27.0 %)	21 (28.4 %)	29 (39.2 %)	3 (4.1 %)	1 (1.4 %)	1.24	0.948
	Not packed	35 (47.3 %)	33 (44.6 %)	6 (8.1 %)	0	0	0.61	0.637

There was a barely significant difference between different grades of ecchymosis on the first day after the procedure (Chai-square; *p* <0.04), but no significant difference in severity was observed between the packed and unpacked sides (Mann–Whitney U; *p* <0.751). However, on the 4th and 7th days post-operatively, significant difference in severity of ecchymosis within the same groups (Chai square <0.01) and between the packed and unpacked groups was be observed (Mann–Whitney U; *p* <0.01). On the 4th day after the operation, more than one half of the unpacked sides (59.6 %) showed ecchymosis of grade 1 or less compared with only 13.5 % on the packed sides. On the 7th day post-op, both sides showed improvement to grade 1 or less in 91.9 and 55.4 % of unpacked and packed sides, respectively. No statistically significant difference in the severity of ecchymosis was observed between external and endonasal rhinoplasty approach at any given time after the procedure (Mann–Whitney U for the 1st, 4th and 7th days post operatively; *p* <0.771, *p* <0.533 and *p* <0.899, respectively).

## Discussion

Periorbital swelling and ecchymosis can adversely influence patient satisfaction after rhinoplasty as it may increase the “down time” after the surgery, and while it may not be apparent immediately after surgery, complete resolution may take up to 2 to 3 weeks. For the most part, lateral osteotomies are to blame for the ecchymosis due to injury to the angular vessels crossing the osteotomy site and from bleeding fractured bone edges. Blood then trickles into the periorbital area and collects under the thin, lax skin of eyelids. Factors that may exacerbate edema and ecchymosis include high osteotomy placement, vigorous rasping of nasal bones and using excessively large or blunt osteotomes [12, 13]. Many technical measures can be instituted to decrease the incidence and severity of post-operative edema and ecchymosis; these include the use of sharp small osteotomes [12, 14], preservation of the periosteal attachment [7], cold compresses [10], and the possible use of a looped drainage tube [15]. In addition, administration of perioperative steroids [9, 16] and remifentanil with controlled hypotension may further contribute to lessen periorbital edema and ecchymosis [17]. Ineffective measures, despite being widely practiced, include infiltration with lidocaine-adrenaline combination [1, 18] and administration of arnica [11].

In this study, another co-factor which may contribute to worsen the postoperative periorbital ecchymosis, nasal pack application, was explored. Despite being unnecessary for most cases [19], nearly one third of rhinoplasty surgeons continue to regularly employ packing following rhinoplasty [20, 21]. Kara et al. reported subconjunctival ecchymosis following rhinoplasty in almost 20 % of their patients, whom were packed bilaterally for a couple of days [22]. In their series, ecchymosis resolved in 11.2 days on average compared with 7 days on the unpacked side in the majority of our patients.

This study provides another reason to limit the use of intra-operative nasal packs unless they are an absolute necessity. Beside the discomfort they impose upon the patient, they increase the odds of developing periorbital ecchymosis. This is most likely due to the accumulation of more blood in the osteotomy site instead of it being drained into the nasal cavity, forcing it into the skin and soft tissues of the periorbital region [15].

A potential limitation of this study is that it only included the patients operated by a single surgeon and that only two raters assessed the outcome. Suggestions to overcome this include enrolling the data from multiple surgeons, utilizing different osteotomy techniques, and additional observers. Their might also be a questionable impact of packing one side on the contralateral side in a given patient, and so a further study utilizing packing on both sides in some patients and none in others might provide additional support to the findings.

## Conclusion

Periorbital ecchymosis after rhinoplasty is influenced by many factors aside from the osteotomies. This study suggests that intra-operative nasal packing plays a significant role in the resultant ecchymosis and it should be only used when necessary; however, larger studies are required to further validate this conclusion. Other measures to reduce edema and ecchymosis such as modifying the surgical technique, cold compresses, and steroids should be used if appropriate in an attempt to decrease the post-operative “downtime” for all aesthetically conscious rhinoplasty patients.
